# Feasibility of Delivering an on-Campus Food Distribution Program in a Community College Setting: A Mixed Methods Sequential Explanatory Investigation

**DOI:** 10.3390/ijerph182212106

**Published:** 2021-11-18

**Authors:** Daphne C. Hernandez, Sajeevika S. Daundasekara, Quenette L. Walton, Chinyere Y. Eigege, Allison N. Marshall

**Affiliations:** 1 Cizik School of Nursing, University of Texas Health Science Center at Houston, Houston, TX 77030, USA; saumali88@gmail.com (S.S.D.); allison.n.marshall@uth.tmc.edu (A.N.M.); 2 Graduate College of Social Work, University of Houston, Houston, TX 77004, USA; qwalton2@central.uh.edu (Q.L.W.); ceigege@uh.edu (C.Y.E.)

**Keywords:** dietary intake, focus groups, food insecurity, food pantry, fruits and vegetables, nutrition, program use

## Abstract

Despite community college students experiencing food insecurity there has been a dearth of research conducted on the feasibility of providing a program designed to increase access to fruits and vegetables among community colleges. This study used a mixed methods sequential explanatory design to examine the feasibility of delivering an on-campus food distribution program (FDP) to community college students and to examine the association between FDP and food insecurity and dietary intake. The study also explored the student’s experiences related to barriers and facilitators of program utilization. In phase one, the FDP occurred for eight months and students could attend twice per month, receiving up to 60 pounds of food per visit. Online questionnaires were used to collect students’ food security and dietary intake. Among the 1000 students offered the FDP, 495 students enrolled, with 329 students (66.5%) attending ≥ 1. Average attendance = 3.27 (SD = 3.08) [Range = 1–16] distributions. The FDP did not reduce food insecurity nor improve dietary intake. In phase two, a subsample of students (*n* = 36) discussed their FDP experiences through focus groups revealing three barriers limiting program utilization: program design and organization, personal schedule and transportation, and program abuse by other attendees. Facilitators to greater program utilization included: the type of food distributed and welcoming environment, along with allowing another designated individual to collect food. To maximize program use, it is suggested that reported barriers be addressed, which might positively influence food insecurity and dietary intake.

## 1. Introduction

The open admission policy and geographic proximity to home, along with the low tuition compared to four-year institutions, make community colleges accessible and a more affordable entry point to higher education in the U.S. Community colleges disproportionally enroll students from low-income backgrounds and who are first-generation, Hispanic or Black, and thirty years or older compared to four-year institutions [1]. These same individuals are at higher risk of experiencing food insecurity [2], which is the lack of reliable access to healthy food due to insufficient money and/or other resources needed to access food [3]. It is estimated that 14% of adults in vocational school and 17% of adults in two-year colleges experience food insecurity [2].

Food insecurity is associated with lower academic performance among community college and university students [4,5,6]. Food insecurity is associated with poor nutrition [7,8] and consumption of less fruits and vegetables [8,9,10,11]. Healthier diets, especially ones that emphasize fruits and vegetables, have been associated with academic achievement among school-aged children [12,13]. Thus, poor diet quality may reduce academic performance among community college students. Poor academic performance is associated with students dropping out of college [14]. Not completing a degree increases the probability that a student will default on their student loans [15,16]. Therefore, reducing food security is important to ensure that more community college students complete their degrees and have a higher probability of paying back student loans.

To reduce food insecurity and to improve access to healthy food among college students, universities are opening on-campus food pantries or cupboard programs. These are food assistance services that collect food donations from the community or receive food from a local food bank. Food is typically organized on shelves and baskets, and students in need select food items. However, campus-based food pantries have limited utilization among university students. According to El Zein and colleagues [17] among 899 students attending the University of Florida that completed the online survey, about 70% of students were aware of the existing on-campus food pantry and almost one-third of survey respondents experienced food insecurity. However, only 38% of food insecure students reported using the on-campus food pantry. In a separate study, low utilization of the on-campus food pantry was observed among students who expressed experiencing food insecurity and attended a mid-sized university in Appalachia [18]. Barriers for use of on-campus food pantries included social stigma/embarrassment, others needing it more, insufficient information on pantry use policies/not knowing how to ask for help accessing food, self-identity, and inconvenient hours [17,18].

Compared to four-year institutions, less research has been conducted on the feasibility of providing a program designed to increase access to fruits and vegetables among community colleges. The Houston Food Bank designed an innovative on-campus food distribution program (FDP) that provides eligible college students access to healthy food. This program targets lower-income students who may be at risk of food insecurity and attempts to reduce food insecurity among college students by providing food directly to students in a discreet and supportive manner. The FDP is based on social cognitive theory. The social-cognitive framework recognizes that individuals, peers/family, and environmental factors influence health behaviors. The FDP provides students with instrumental support to indirectly increase educational self-efficacy. Specifically, the observational learning and modeling (i.e., seeing other students at the FDP) and reinforcements (i.e., consistent access to food) could potentially facilitate students to stay in school and complete degree requirements [19]. 

The purpose of the current study was to assess the feasibility of delivering an on-campus FDP using a mixed-methods sequential explanatory design [20] and a framework outlined by Orsmond and Cohn [21]. In phase one, we (1) demonstrate the feasibility of recruiting community college students who experience low income to participate in an intervention (2) demonstrate the feasibility of data collection procedures, and (3) examine poundage of food received, dietary intake, and food insecurity. In phase two, focus groups were used to examine participants’ acceptability responses to the intervention in relation to barriers and facilitators to utilizing the program. The study contributes to the literature that has primarily focused on food insecurity among university students by concentrating on a *community college* sample. From a practical perspective, the findings may assist college administrators and community partners when designing and delivering an on-campus FDP to serve community college students who experience low income. 

## 2. Materials and Methods

### 2.1. Setting

Students based on the criteria listed below were recruited for the study from two campuses within a large community college system that operates community colleges throughout Houston and the greater Houston area. The two campuses that were selected had the space needed to deliver the FDP. The community college system does not have on-campus housing available for its students. Enrolled students are primarily Black and Hispanic adults, over the age of 25, and attend part-time. Over half of the students qualify for the federal Pell Grant. The cohort default rate in 2017 was 13.7% [22].

### 2.2. Study Design and Eligibility

**Figure 1:**
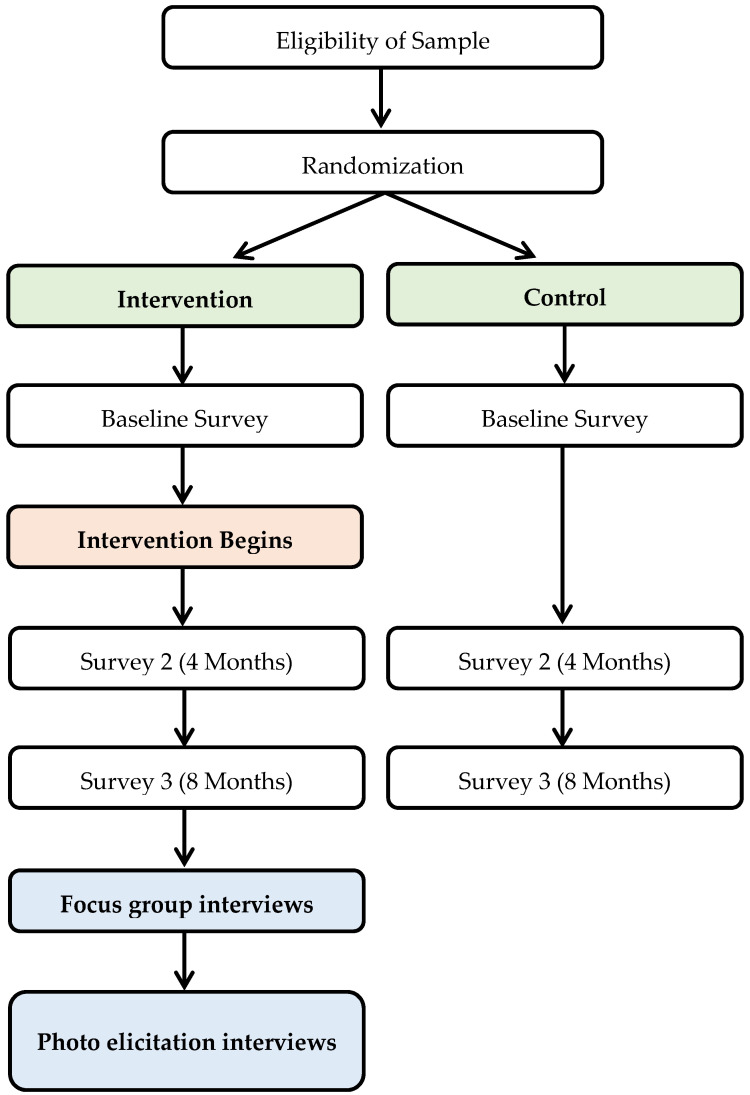
Food Distribution Program Study Flow Chart.

Using a mixed-methods sequential explanatory design [20], the study began with collecting data from a randomized control intervention (phase 1) and followed up with focus group and photo-elicitation interviews (phase 2). The flow of the study can be found in Figure 1.

The study was designed to have 1000 students receive the intervention; however, to assist with program manageability for involved community partners, it was structured as two cohorts of 500 students going through the 8-month intervention at a time. Five hundred students were randomly selected in January 2018 and another set of 500 students were randomly selected in August 2018. An additional 500 students were randomly selected at both time points to serve as a control group (Supplementary Figures S1 and S2).

A community college staff member identified eligible students using the college’s administrative records. Part-time and full-time students who listed one of the two campuses selected for the intervention as their primary campus of enrollment were eligible. In addition, students had to be 18 years or older, and have an expected family contribution of $0 and total income reported in federal student aid forms of $25,000 or less. For continuing students, satisfactory academic progress as determined by the community college was also a requirement. Students who were 17 years of age and those who were concurrently in high school were excluded.

### 2.3. Randomization

The data was organized (blocked) based on students’ sex. Within each block, students were randomly allocated in a 1:1 ratio by a community college staff member to either the treatment or control group using a computerized random number generator. Baseline checks were performed to ensure the sample met the criteria for successful randomization. Students who were randomly assigned to the control condition had access to all standard community college services except the FDP.

### 2.4. Recruitment for the Intervention

Students assigned to the intervention were notified via email that they were a recipient of a food scholarship, and they could access up to 60 pounds of perishable food items (fruits, vegetables, meat) and non-perishable food (dry goods) per on-campus food distribution visit at no cost. They could visit the distribution twice a month, and distributions occurred on two of the community college campuses. The email also contained a link to accept the invitation to participate in the program, along with a link that contained the food distribution schedule. In addition, the email included a video of a community college student explaining the benefits of the program. To assist with making students aware of the program, the community college sent students a text message about checking their email regarding a scholarship that they had qualified for. The community college’s call center also notified the students assigned to the intervention about the scholarship.

### 2.5. Survey

In phase one of the study design [20], all students in the treatment and control group were requested to complete an online survey prior to launching the intervention program (S1), during the program (S2), and at the end of the program (S3). The survey contained various measures of material hardship (including food insecurity), public assistance program participation, dietary intake, major life events, self-efficacy, sense of belonging, stress, and mental health. The students were invited through an emailed link to complete the online survey that was managed by a third party. Students were compensated for their time and effort for completing the survey at each time point in the form of a $25 electronic gift card that was also managed by a third party. The community college provided the student-level administrative data on demographics, grade point average, credits attempted, and credits completed.

### 2.6. Intervention: Food Distribution Program

**Figure 2:**
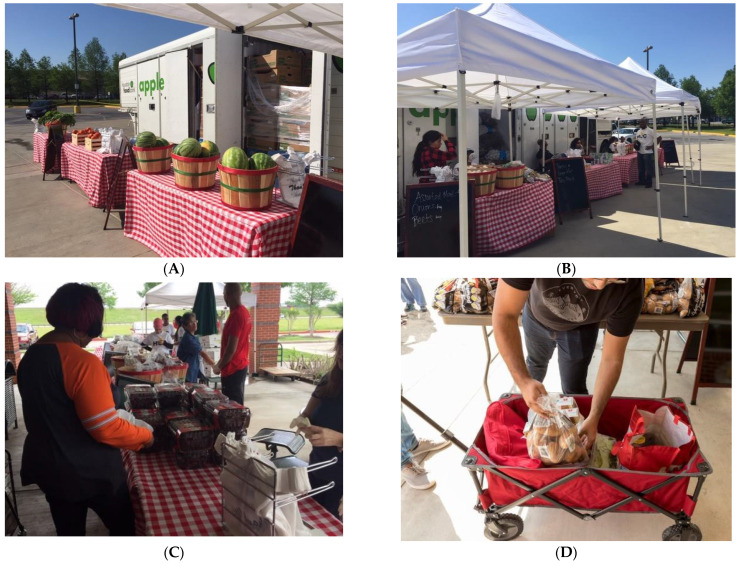
Pictorial of the On-campus Food Distribution Program. (**A**,**B**) demonstrates how the program was set up similar to a farmer’s market. (**C**,**D**) displays students selecting their own food items (“client’s choice”).

Food distributions occurred on-campus once per week, lasting for four hours each, on alternating Wednesdays and Fridays. Students were given the opportunity to identify up to two persons who could serve as a “substitute shopper” on their behalf to collect food if they were unable to attend. Each week food was set up similar to a farmer’s market experience, and students were allowed to select their own food items (“client’s choice”) (Figure 2).

### 2.7. Focus Groups and Photo Elicitation Interviews

In phase two of the study design [20], qualitative methods—a combination of focus groups and photo-elicitation interviews—were conducted to explore in-depth results from the survey findings. After the conclusion of FDP, students who accepted the invitation to participate in the program were categorized as no/low attendees (attended 0–2 distributions) vs. high (attended ≥ 3 distributions) based on their total number of visits to the food distributions. Students who had completed at least one survey were invited to participate in a follow-up focus group and photo-elicitation interviews (January 2020 to April 2020). A semi-structured focus group interview guide was developed by the research team and used to facilitate the discussion regarding barriers and facilitators for program utilization. The focus groups were conducted in English by two researchers (D.C.H and Q.L.W.) trained in qualitative interviewing and experienced in working among this population. Separate focus group discussions were conducted for no/low attendees and high attendees. Focus groups were audio-recorded and transcribed verbatim. Participants received an additional $25 electronic gift card as compensation for their time. Further details about the focus group procedures have been previously reported [23].

Individuals that participated in the focus groups were invited to participate in the photo-elicitation interviews. Photo elicitation interviews were conducted to gain a more in-depth understanding of food access challenges and the methods have also been previously reported [24]; however, the current study focuses on the findings from the focus group. Data collection was approved by the Institutional Review Board at the University of Houston; data analyses were approved by the Institutional Review Board at the University of Texas Health Science Center at Houston.

### 2.8. Measures

#### 2.8.1. Recruitment Capability and Resulting Intervention Sample Characteristics

Student enrollment into the program was recorded by the community college staff. Administrative data were used to extract students’ age (in years based on birth year), gender, race/ethnicity (non-Hispanic White, non-Hispanic Black, Hispanic and other race), marital status (married, divorced/separated, single), current academic level (freshman, sophomore, associate degree, bachelor’s degree, master’s degree, unclassified/not available), and employment status (full-time employed, part-time employed, unemployed).

#### 2.8.2. Data Collection Procedures and Outcome Measures

The number of students that attended the weekly food distributions, who attended (student vs. substitute shopper), and the poundage of food distributed were recorded each week by trained research assistants. In addition, outcomes measures (food security and nutrition) were collected through survey measurements. *Food security*. The 18-item Food Security Module developed by the United States Department of Agriculture (USDA) assesses the quality and quantity of food consumed over the past 30 days (e.g., “Over the last 30 days, the food that I bought just didn’t last and I didn’t have money to get more). The students were asked to rate how true each statement was using a 3-point Likert scale (1 = Often true, 2 = Sometimes true, 3 = Never true), by selecting yes/no, or providing the number of days with such experiences. Responses of “often,” “sometimes true,” “yes,” and “3 or more days” were coded as affirmative responses. Based on the USDA cutoff criteria, households were considered food insecure if they provided three or more affirmative responses [25]. *Dietary intake*. Student dietary intake was assessed using the validated Block rapid food screener for fruits-vegetables-fiber and screener for dietary fat [26]. The Block rapid food screener is a short self-administered tool, with 10-items for fruit and vegetable intake and 17-items for meat and snack intake. Each item asked the responders how often they consumed the listed food over the past year. Items were summed to create a fruit-vegetable score, a fruit-vegetable-bean score, and a meat/snack score. The equations used to calculate the fruit/vegetable servings, dietary fiber, and micronutrients, along with equations to estimate total fat, saturated fat and dietary cholesterol can be found in Supplementary File S1.

#### 2.8.3. Participants’ Acceptability and Responses to the Intervention

Each focus group discussion began with an introduction of the study providing participants information related to the purpose of the focus group. The focus group questions were designed to evaluate the facilitators and barriers of participation in an on-campus FDP on the dietary intake of community college students. We asked about (a) knowledge about the FDP; (b) experience with the FDP, and (c) barriers and facilitators to accessing the FDP. We also used paraphrasing and probing questions to gather further information regarding the topic being asked and to gain deeper meanings about the participants’ experiences.

### 2.9. Data Analysis

Descriptive data were analyzed using Stata version 16.0 [27]. Summary statistics are reported as means and standard deviations (SD) or frequencies and percentages. Independent sample *t*-tests or proportion tests were used to determine socio-demographic differences between students that enrolled in the program and students that never enrolled. Similar comparisons were made between no/low attendees and high attendees. Paired *t*-tests or proportion tests were used to determine significant differences from S1 to S3 in food security categories and dietary intake among no/low attendees and high attendees. As dietary recommendations are age- and sex-specific [28], the dietary data were analyzed separately for females and males.

The focus group data were examined using the constant comparative approach of grounded theory [29] using Dedoose version 8.3.43 [30]. First, the research team read each focus group transcript reflecting upon initial thoughts and memos written after each group. The focus group was considered the unit of analysis instead of each individual in the groups as separate cases [31,32,33]. Second, the research team re-read each focus group transcript to develop codes for our codebook. Once the codebook was developed, a postdoctoral scholar and a graduate-level research assistant independently coded the focus group transcripts. Discrepancies with the codes were discussed in the research team meetings until there was a consensus. Third, we identified emerging themes using relevant concepts found in the literature regarding community college students’ experiences with food insecurity [34,35]. Further, we identified additional themes that emerged from the data itself.

To ensure consistency in the codes, an inter-rater reliability test for the initial round of coding was conducted (*r* = 0.69). Initial coding of a random sample of transcripts was first performed and then a pooled Cohen’s Kappa test in Dedoose was conducted to test inter-rater reliability; the subsequent coder took the test. These two coders reviewed areas of agreement and as a team, we discussed and reconciled areas of disagreement. Once all data were coded, themes were compared across the dataset and then reviewed for comprehensiveness to finalize analyses.

#### Trustworthiness and Credibility

Several steps were taken to ensure the credibility of the findings and to increase the trustworthiness of our research findings [36,37]. First, credibility was established through frequent research team meetings [37]. The research team meetings provided the opportunity to test developing ideas and interpretations of the data to ensure that the researchers were not biasing the results. Second, memos and reflexive journals were produced to monitor personal biases, expectations, and assumptions about the data and emerging themes. Third, multiple coders were used to enhance the trustworthiness of the study. Last, elaborate descriptions to support the emerging themes were produced [37,38].

## 3. Results

### 3.1. Recruitment Feasibility and Resulting Intervention Sample Characteristics

Supplementary Table S1 indicates that there were no differences in terms of demographics and academics between cohorts 1 and 2. For this reason, the cohorts were combined in analyses. As the objective of the current study is to report on the feasibility of the program, the findings below are focused solely on the sample that was offered the program.

The demographic characteristics of the total sample of students offered the FDP is provided in Table 1. Among the group of students offered the FDP, 66.9% were female, aged 18–25 years (47.9%), 54.4% were non-Hispanic Black students and 84.5% were single. The majority of those who were offered the program were first-year students (72.4%).

### 3.2. Evaluation of Data Collection Procedures and Sample Characteristics of the Students That Enrolled

**Figure 3:**
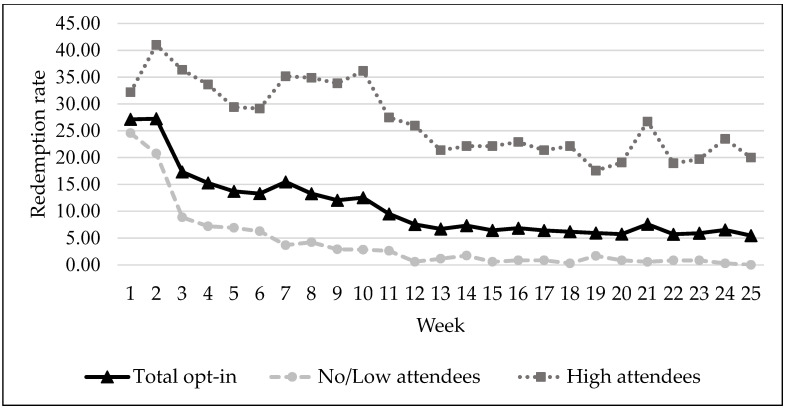
Rate of food scholarships redemption of the students enrolled into the program (*n* = 495) and by the level of attendance at each week of food distribution over the 8 months duration (over 25 weeks of food distribution). Note. The weekly redemption rate was calculated as the percentage of students that attended the distribution in relation to the total number of students enrolled each week.

A total of 25 food distributions were conducted during the eight months, and students were eligible to attend a maximum of 16 distributions. The scholarship redemption rate at each week of food distribution among students that enrolled and by the level of attendance is illustrated in Figure 3. The average weekly redemption rate was 10.68 (SD = 6.06) indicating that on average about 11% of the students that enrolled in the program attended the distribution each week. The maximum redemption rate was 27.24% at week two and the lowest was 5.45% at week 25.

**Figure 4:**
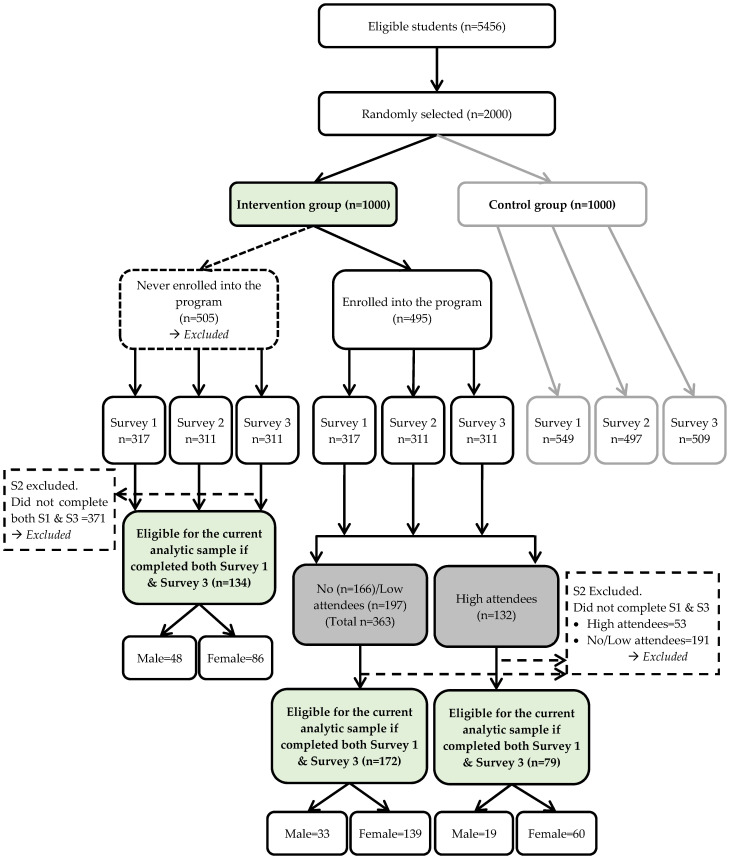
Consort diagram of intervention group based on completion of both survey 1 and survey 3. Note. S1 = Survey 1; S2 = Survey 2; S3 = Survey 3.

A total of 495 (49.5%) students enrolled in the program (Figure 4), and demographic characteristics by level of attendance are displayed in Table 1. Students who enrolled were significantly older (t = −6.10, *p* < 0.001), more likely to be female (z = −5.62, *p* < 0.001) and married (z = −1.98, *p* = 0.048) compared to the students who never enrolled. The high attendees were significantly older compared to no/low attendees (t = −4.96, *p* < 0.001). There were significantly more students of the other race/ethnicity (including Asians and Native Americans) category among the no/low attendees compared to high attendees (z = 2.42, *p* = 0.016). There were no other significant differences among the demographic characteristics between the no/low and high attendees. Among high attendees, 70.5% (*n* = 93) of students had listed a substitute shopper, while only 28.7% (*n* = 104) of no/low attendees had a substitute shopper (results not shown).

**Figure 5:**
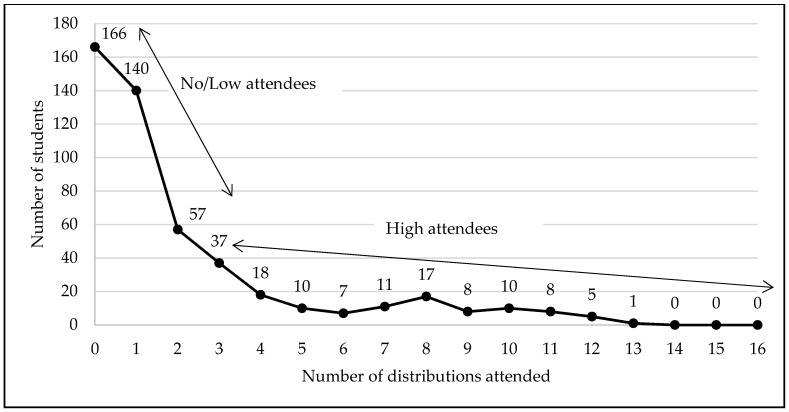
Frequency of visits: Number of distributions attended by the number of students among the students enrolled in the program (*n* = 495). No/low attendees visited the food distribution 0–2 times, and the high attendees visited the food distribution 3–16 times.

When examining the redemption by frequency of visits, among the 495 students that enrolled, 166 (33.5%) did not attend any food distributions and 329 total students (66.5%) attended at least one food distribution (Figure 5). Among the 329 students that attended the distributions, 197 students (39.8%) visited one/two food distributions and 132 students (26.7%) attended ≥ 3 distributions. Overall, given the entire treatment group (*n* = 1000), 329 students (32.9%) attended the distribution once and 671 (67.1%) of the students never attended the distribution.

Among students enrolled in the program (*n* = 495), mean attendance (i.e., utilization) was 2.17 (SD = 2.95) distributions and the maximum attendance was 13 distributions [possible range = 0–16]. Average attendance among no/low attendees (0–2 visits) was 0.70 distributions (SD = 0.73) and average attendance among high attendees (≥3 visits) was 6.22 (SD = 2.96). Among students that attended at least one distribution, mean attendance was 3.27 (SD = 3.07; possible range 1–16) distributions (results not shown).

### 3.3. Evaluation of Outcome Measures

#### 3.3.1. Poundage of Food

**Figure 6:**
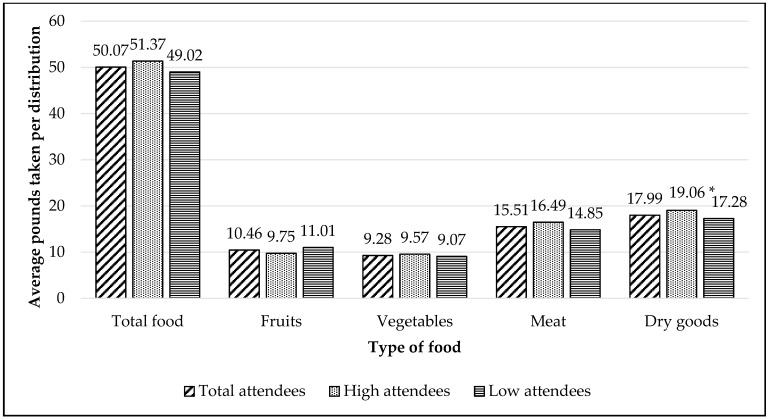
Average pounds of food students received per distribution by all attendees (*N* = 329) and by two levels of attendance (*n* = 132 high attendees; *n* = 197 low attendees). Note. * A significant difference in the poundage taken by high attendees compared to low attendees at *p* < 0.05.

The following averages are based on students who attended the food distribution program (*n* = 329). Per visit, students averaged 50.07 lbs. (SD = 15.24) of total food, including 10.46 lbs. (SD = 7.49) of fruits, 9.28 lbs. (SD = 4.80) of vegetables, 15.51 lbs. (SD = 8.35) of meat and 17.99 lbs. (SD = 7.61) of dry goods (Figure 6). High attendees received on average 51.37 lbs. (SD = 9.09) of total food and low attendees received 49.20 lbs. (SD = 18.20) of total food per visit. High attendees were significantly more likely to take higher amount (pounds) of dry goods (t = 2.098, *p* = 0.037) compared to low attendees. However, there were no significant differences in the fruit (t = −1.442, *p* = 0.150), vegetable (t = 0.914, *p* = 0.361), meat (t = −1.745, *p* = 0.082), or total food (t = 1.268, *p* = 0.206) selected by the high attendees in comparison to low attendees.

#### 3.3.2. Dietary Intake

The dietary data for students that enrolled (no/low attendees and high attendees) and students that did not enroll at two time points (S1 and S3) by sex are reported in Table 2 with the micronutrient and fat intake recommendations from the Institute of Medicine [28]. Among female students, the dietary fiber value at S1 was significantly lower among high attendees compared to no/low attendees (t = 2.085, *p* = 0.038) and the students that never enrolled (t = −2.947, *p* = 0.004). Further, high attendees had a significantly lower fruit/vegetable/bean score (t = −2.071, *p* = 0.040), vitamin C (t = −2.423, *p* = 0.017), magnesium (t = −2.726, *p* = 0.007) and potassium (t = −2.627, *p* = 0.010) values at S1 compared to students that never enrolled. At S3, high attendees had a significantly lower meat/snack score (t = −2.933, *p* = 0.004), total fat (t = −2.933, *p* = 0.004), percent fat (t = −2.933, *p* = 0.004), saturated fat (t = −2.933, *p* = 0.004), and dietary cholesterol (t = −2.904, *p* = 0.004) value compared to the students that never enrolled. Further, high attendees continued to have a lower dietary fiber value compared to students that never enrolled (t = −2.155, *p* = 0.033).

However, among female students, none of the groups showed a significant change in fruit/vegetable score (never enrolled: t = 1.690, *p* = 0.095; no/low attendees: t = 1.822, *p* = 0.071; high attendees: t = 0.440, *p* = 0.662), fruit/vegetable/bean score (never enrolled: t = 1.404, *p* = 0.164; no/low attendees: t = −0.436, *p* = 0.664; high attendees: t = 0.259, *p* = 0.796) or meat/snack score (never enrolled: t = 1.199, *p* = 0.235; no/low attendees: t = 0.165, *p* = 0.869; high attendees: t = 1.199, *p* = 0.235) resulting in no significant changes in micronutrient or fat intakes.

There were no differences in S1 or S3 dietary scores between the three groups among male students, except for dietary fiber intake at S3. The high attendees had a significantly lower dietary fiber values compared to students that never enrolled (t = −2.015, *p* = 0.048). Further, among male students, none of the groups showed a significant change in fruit/vegetable score (never enrolled: t = −1.448, *p* = 0.154; no/low attendees: t = −0.461, *p* = 0.648; high attendees: t = 0.912, *p* = 0.374), fruit/vegetable/bean score (never enrolled: t = −1.389, *p* = 0.171; no/low attendees: t = −1.005, *p* = 0.322; high attendees: t = 0.603, *p* = 0.554) or meat/snack score (never enrolled-in: t = −0.685, *p* = 0.497; no/low attendees: t = 0.070, *p* = 0.945; high attendees: t = 0.709, *p* = 0.488) resulting in no significant changes in micronutrient or fat intakes.

#### 3.3.3. Food Insecurity

Among the 1000 students offered the program only 385 (38.5%) completed both the baseline (S1) and final (S3) surveys, including 251 enrolled students and 134 students who never enrolled (Table 3). The sample of students who completed both surveys were significantly more likely to be female (z = −3.789, *p* < 0.001), Hispanic (z = −1.969, *p* = 0.049), and divorced/separated (z = −2.049, *p* = 0.041) compared to students excluded due to not having survey data (results not shown). The rate of food insecurity at baseline was not significantly different between students that enrolled and students that never enrolled. (z = −1.710, *p* = 0.087). Among the 251 students that enrolled in the program and completed both surveys, 172 were no/low attendees and 79 were high attendees. There was also no significant difference in the rate of food insecurity at S1 between the low and high attendees (z = −0.376, *p* = 0.707).

By the end of the FDP, the food insecurity rate (z = −4.055, *p* < 0.001), and specifically the very low food security rate (z = −2.834, *p* = 0.005) were significantly higher among students that enrolled compared to students that never enrolled. However, food insecurity rates were not significantly different between the no/low and high attendees at the final time point (S3).

From S1 to S3 there were no significant change in the rates of food insecurity among students that enrolled (z = −0.468, *p* = 0.640) nor in students that never enrolled in the program (z = 1.710, *p* = 0.087). Examining the severity of food insecurity from S1 to S3, there was no significant change in rates of low food security and very low food security among either the no/low attendee group or the high attendee group. The low food security rate significantly decreased among the students that never enrolled in the program between the two-time points (z = 2.269, *p* = 0.023) (results not shown).

### 3.4. Evaluation of Participants’ Acceptability and Responses to the Intervention

**Figure 7:**
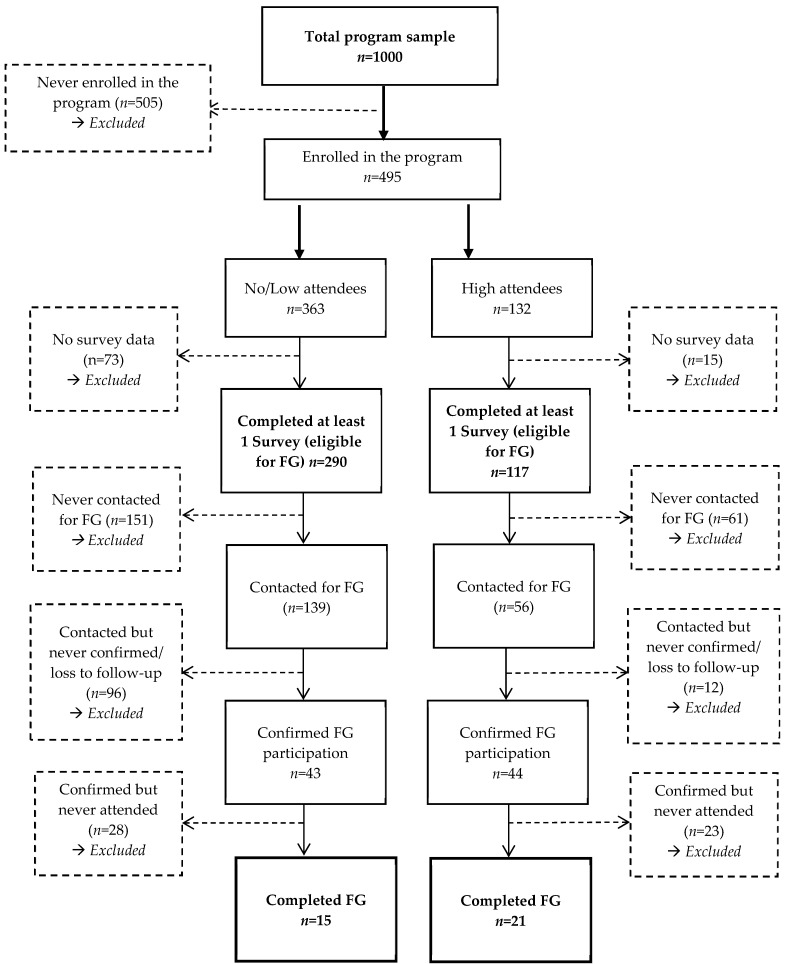
Consort diagram of focus groups (FG).

A total of 36 participants shared their experiences related to the barriers limiting their utilization of FDP and facilitators that improved their FDP participation (Figure 7).

### 3.5. Barriers Limiting FDP Utilization

Based on the focus group discussions among no/low attendees and high attendees, three main barriers emerged, which were experienced by both groups of students: 1. The program’s design and organization, 2. Personal schedule and transportation, and 3. Program abuse by other attendees. The following section explains these barriers in detail.

#### 3.5.1. The Program’s Design and Organization

For some students, the program organization including the check-in and check-out process was discouraging. In some instances, students had to wait in line for an extended length of time to complete the check-in and check-out process (weighing the food). Most students stated that it would be easier for them if this process was faster, shorter, or if there were further assistance for students with medical needs. For the majority of students, this was the main reason for not attending the maximum number of distributions they were eligible to attend.

#### 3.5.2. Personal Schedule and Transportation

In addition to the barriers related to the FDP, many students talked about personal barriers that hindered their program utilization. The most commonly stated personal barriers included personal schedules (including school and work schedules) and transportation. Students explained how the program schedule did not match their personal schedule. Students explained how the FDP was designed to have food distributions on only certain days of the week for a limited time period at only two campus locations. This was a problem for some students because even though they were enrolled in the campus selected for the food distribution, their place of residence or work was much further from the campus location. Some students had conflicting obligations during the days/times of the distributions, including attending classes at the same or different campuses, or attending to work responsibilities. Further, since the food distributions were held only at one location out of the two pre-selected locations each week and it was the same day and time of the week for each location, if a student missed a food distribution there were no other alternatives to receive food for that week unless they had a “substitute shopper” (see facilitators below). For some, transportation was another personal challenge that had to be overcome to attend the FDP including not having a personal vehicle and having to use public transportation to attend the distribution. Some participants did not attend FDP because it was hard for them to carry food on public transportation and some could not find a person to drive them to and from the distributions. There were also a few students who did not drive and that was their reason for not attending the FDP.

#### 3.5.3. Program Abuse by Other Attendees

Many students discussed how the behavior of some of the other participants affected their decision to not participate in the FDP. Even though it was not a regular occurrence, some students shared their experiences of observing some participants taking too much food or too much of certain foods, such that it limited the amount of food/variety of food available for other participants. Specifically, some students talked about how they were unable to receive much food (especially meat) if they arrived even 30 minutes after the food distribution started, due to participants attending before them taking more than their fair share of each food item. Most of the students agreed that this was one of the reasons for them to not attend FDP or not take the maximum allowed amount of food even when they did attend the food distributions.

### 3.6. Facilitators for FDP Utilization

Among focus group data from both no/low attendees and high attendees, two types of facilitators emerged: 1. Type of food distributed and welcoming environment and 2. Allowing a substitute shopper to collect food. These themes were discussed only among focus group participants with at least a single visit to food distributions and mainly among high attendees.

#### 3.6.1. Type of Food Distributed and Welcoming Environment

Students talked positively about the type of food distributed at the program, the volunteers/staff attending the FDP, and the organization of the program. These were also described as facilitators to greater participation in the program. For most of the students, providing fresh/canned fruits, fresh/canned vegetables, and meat at the distribution was a leading factor for attendance. Students talked about how these food items were generally more expensive and being able to receive them at the distribution made them more likely to attend distributions as much as they could. They also talked about how the volunteers/staff attending the FDP treated them and made them feel welcome. Some compared the FDP to other food assistance programs that they utilized and explained how it was easier for them to collect food at the FDP. They also expressed that the experience was less chaotic compared to other community food distributions they had attended.

#### 3.6.2. Allowing a Substitute Shopper to Collect Food

Many focus group participants in the high attendance group (71%) had at least one substitute shopper listed compared to 20% of no/low attendees. The high attendees talked about the advantage of the program allowing them to use a substitute shopper to collect food on their behalf. Most participants discussed how their family members, friends, or roommates went to collect food when they were unable to do so. This allowed them to receive food despite the barriers they discussed. Students agreed that if the program did not allow someone else to collect food, it would be a greater challenge for them to attend the FDP, and they were grateful for having this option in the program. A summary of quotes/examples from students supporting each theme discussed above are found in Table 4.

## 4. Discussion

### 4.1. Recruitment Feasibility

Guided by the feasibility framework outlined by Orsmond and Cohn [21], the current study assessed the feasibility of delivering an on-campus FDP using a mixed-methods sequential explanatory design [20]. Our results indicate that it is feasible to recruit community college students who experience low income to participate in an on-campus FDP. Greater recruitment efforts were placed at the beginning of the program, which is reflected by the high redemption rates in the first two weeks of the program. The decrease in redemption rate over time is similar to other programs intended to increase fruit and vegetable intake and decrease food insecurity [39]. To increase enrollment, it may be necessary to sustain recruitment efforts throughout the program. In addition, the recruitment efforts must provide clear and reinforcing messaging throughout. In the end, 50% of the recruited sample enrolled in the program. The program appears to appeal or be more feasible to students that are older, female, and married. A high percentage of female participation has been observed in other nutrition programs intended to increase fruits and vegetables [39,40,41,42].

### 4.2. Utilization

One-third of the sample selected for the program attended. A greater percentage of adults who frequented the program more often (termed “high attendees”) were older. The focus groups were extremely valuable in helping to contextualize the quantitative findings. For instance, several of the participants in the focus group discussed how when they were younger, pride and the social stigma associated with public assistance programs prevented them from asking for or accepting help. Having reflected on their personal struggles, high attendees were no longer ashamed to receive assistance but stated that they believed this could be why other, younger students, may have not attended. High attendees were also more likely to list a substitute shopper, which was reflected in the focus groups as a great asset of the program. While the substitute shopper was not always the individual picking up the food, the ability to name someone that could come to the distribution on their behalf suggests that some students had greater instrumental or tangible social support than others. Instrumental or tangible social support involves individuals directly assisting others. The ability to rely on trusted individuals has been demonstrated to be impactful when promoting health [43,44]. While the primary focus of the intervention was not on health, the program indirectly promoted health by providing community college students access to fruits and vegetables. Thus, it appears that high attendees were less concerned about the potential stigma associated with receiving food assistance and had more instrumental (or tangible) social support compared to low attendees. Both of these characteristics may have contributed to their ability to access the program more often.

Similar to other campus-based food assistance programs, this program had low utilization [17,18]. While it was originally perceived that an on-campus distribution would be the most beneficial for students, this was not always the case for several reasons. The study design relied on the campus location the students enrolled for classes; however, the location of enrollment was not necessary the campus location where students received their courses. Courses may have been offered at other campus locations based on their major or offered online. The campuses are located throughout a large metropolitan area, making it difficult to attend the FDP. The physical distance between the FDP and work or home was also perceived as a barrier by the student and reflected in the focus groups. Low attending students and students that were not able to attend discussed how not having access to transportation also deterred their participation. Transportation has been observed as a barrier to food access among adults who experience low income and food insecurity [45,46,47,48,49].

### 4.3. Dietary Intake

A similar amount of food (as measured by pounds) was recorded among high and low attendees, except for high attendees choosing more pounds of dry good items compared to low attendees. Because similar poundage was recorded within attendance groups, this may be why there were no significant changes in dietary intake, micronutrients, or fat intake within attendance groups over time. While the focus group discussion did not provide a clear explanation as to why dry goods were sought after, among the high attendees, participants in the focus groups did discuss how perishable items favorably influenced their participation. High attendee females had lower intakes of fruits, vegetables, beans, and micronutrients compared to females that never enrolled in the program, which may reflect a need among high attendee females and why the program was used more frequently among this group. The focus on distributing greater amounts of perishable food items addresses a challenge that food pantries have in providing nutrient-dense foods to clients [50].

### 4.4. Food Insecurity

By the end of the program, a greater proportion of students enrolled in the program experienced very low food security compared to students who never enrolled. Over time, very low food security decreased among students who never enrolled in the program. While this is unexpected, the students that never enrolled may have had other means to obtain food that was more accessible (and addressed barriers discussed in the focus group) that were beyond the purview of the program and this study.

However, among the students that enrolled in the FDP, food insecurity remained the same over time. Despite the program making food available, the program did not address the various programmatic and personal accessibility barriers, including various forms of material hardship, that were revealed in the focus group discussions and have been documented in previous research [51,52]. Some of the barriers were reduced among people that had instrumental support in the form of substitute shoppers; yet, the overarching anxiety of where they were going to get the next meal that coincided with other forms of material hardship were still present.

## 5. Conclusions

While it is feasible to deliver an on-campus FDP to community college students, the average program utilization was low, similar to other programs [17,18]. In addition, the program was unable to reduce food insecurity and improve dietary intake among attendees. This may be because similar poundage of food was recorded within attendance groups. While the FDP made food available to students, the FDP did not address the various forms of material hardship that are correlated with food insecurity and that were described in the focus group discussions [51,52]. The next step, as suggested by others [53], is to conduct a rigorous evaluation to compare food insecurity and dietary intake between the treatment and control groups.

Although Texas has not developed legislation to address food insecurity among college students [54], smaller-scale implementation recommendations for future FDP in a community college setting are as follows. It is recommended that the substitute shopper approach be implemented, which facilitated program participation in the current study. To maximize utilization, it is important to minimize student-reported barriers in accessing FDP. A way to do this is to provide the FDP more frequently on the same campus or provide the opportunity to attend an FDP that is not located on a community college campus but still within a particular geographic area. In addition, it is recommended to implement transportation assistance or a food delivery system to decrease transportation barriers. The implementation of such recommendations may have a positive influence on food insecurity and dietary intake.

## Figures and Tables

**Table 1:** Demographic characteristics of the total program sample and by program enrollment status and level of attendance (*n* = 1000), mean (SD) or frequency (%).

Characteristic	Total Program Sample (*n* = 1000)	Program Sample (*n* = 1000)		Enrolled into the Program (*n* = 495)	
		Never Enrolled into the Program (*n* = 505)	Enrolled into the Program (*n* = 495)	No/Low Attendees (*n* = 363)	High Attendees (*n* = 132)
**Age**	29.65(10.35)	27.71 (9.10)	31.63 (11.15) ^a^	30.16 (10.40)	35.66 (12.16) ^b^
**Gender**					
Male	331 (33.1%)	209 (41.4%)	122 (24.7%) ^a^	91 (25.1%)	31 (23.5%)
Female	669 (66.9%)	296 (58.6%)	373 (75.4%)	272 (74.9%)	101 (76.5%)
**Race/ethnicity**					
Non-Hispanic white	79 (7.9%)	37 (7.3%)	42 (8.5%)	34 (9.4%)	8 (6.1%)
Non-Hispanic black	544 (54.4%)	263 (52.1%)	281 (56.8%)	200 (55.1%)	81 (61.4%)
Hispanic	289 (28.9%)	156 (30.9%)	133 (26.9%)	94 (25.9%)	39 (29.6%)
Other	88 (8.8%)	49 (9.7%)	39 (7.9%)	35 (9.6%)	4 (3.1%) ^b^
**Marital status**					
Married	78 (7.8%)	31 (6.2%)	47 (9.5%) ^a^	35 (9.6%)	12 (9.1%)
Divorced/separated	77 (7.7%)	31 (6.2%)	46 (9.3%)	32 (8.8%)	14 (10.6%)
Single	844 (84.4%)	442 (87.7%)	402 (81.2%) ^a^	296 (81.5%)	106 (80.3%)
**Academic level**					
Freshman	724 (72.4%)	378 (74.9%)	346 (69.9%)	262 (72.2%)	84 (63.6%)
Sophomore	59 (5.9%)	30 (5.9%)	29 (5.9%)	18 (5.0%)	11 (8.3%)
Associate degree	76 (7.6%)	35 (6.9%)	41 (8.3%)	28 (7.7%)	13 (9.9%)
Bachelor’s degree	29 (2.9%)	13 (2.6%)	16 (3.2%)	14 (3.9%)	2 (1.5%)
Master’s degree	5 (0.5%)	3 (0.6%)	2 (0.4%)	1 (0.3%)	1 (0.8%)
Unclassified/not available	107 (10.7%)	46 (9.1%)	61 (12.3%)	40 (11.0%)	21 (15.9%)
**Employment status**					
Full-time employee	114 (11.4%)	59 (11.7%)	55 (11.1%)	39 (10.7%)	16 (12.1%)
Part-time employee	153 (15.3%)	85 (16.8%)	68 (13.7%)	49 (13.5%)	19 (14.4%)
Not employed	733 (73.3%)	361 (71.5%)	372 (75.2%)	275 (75.8%)	97 (73.5%)

Note. Data is based on the student-level administrative data provided by the community college. The comparison test report includes an independent sample *t*-test for continuous variables and probability tests for categorical variables. ^a^ Students enrolled into the program different from those never enrolled in the program, *p* < 0.05. ^b^ No/low attendees different from high attendees, *p* < 0.05.

**Table 2:** The dietary intake of the students at the baseline and the end of the food distribution program compared to recommended intakes by the Institute of Medicine (IOM). Given as mean (SD) by sex and the program enrollment status and level of attendance (included only students completed both baseline and end of program survey and no missing data for dietary variables, *N* = 385).

**Female (n = 285)**							
**Dietary Intake Variables**	**No/Low Attendees (n = 139)**		**High Attendees (n = 60)**		**Never Enrolled (n = 86)**		**Recommended Intakes ^a^**
	**S1**	**S3**	**S1**	**S3**	**S1**	**S3**	
Fruit/vegetable score	11.44 (6.08)	10.40 (6.03)	9.95 (6.21)	9.58 (7.08)	11.93 (7.09)	10.97 (6.29)	NA
Fruit/vegetable/bean score	15.17 (8.43)	13.80 (8.28)	13.05 (7.54) ^f^	12.78 (8.55)	16.16 (9.79)	15.04 (8.73)	NA
Meat/snack score	24.29 (11.72)	24.12 (12.39)	22.45 (11.28)	20.63 (10.84) ^f^	25.73 (11.10)	26.17 (11.50)	NA
**Fruit and vegetable intake**							
Fruit and Vegetable servings (per day)	3.47 (2.23)	3.08 (2.22)	2.92 (2.27)	2.82 (2.55)	3.64 (2.62)	3.30 (2.29)	
**Micronutrient intake**							
Vitamin C (mg)	115.97 (55.86)	106.67 (54.91)	100.92 (49.15) ^f^	98.96 (56.89)	124.05 (64.57)	116.32 (57.99)	75.0
Magnesium (mg)	302.94 (99.55) ^e^	286.10 (97.97)	274.39 (86.29) ^f^	270.53 (101.93) ^f^	320.26 (113.92)	306.07 (103.25)	320.0
Potassium (mg)	2902.53 (979.63)	2737.43 (963.67)	2626.91 (852.92) ^f^	2590.09 (1001.16)	3063.54 (1125.36)	2925.07 (1016.76)	2600.0
Dietary fiber (g)	14.20 (6.72) ^e^	13.06 (6.62)	12.19 (5.78) ^f^	11.93 (6.89) ^f^	15.51(7.61)	14.54 (6.95)	25.0
**Fat intake**							
Total fat (f)	102.19 (28.12)	101.79 (29.74)	97.78 (27.07)	93.42 (26.03) ^f^	105.66 (26.64)	106.72 (27.59)	NA ^b^
Fat %	36.67 (7.03)	36.57 (7.44)	35.57 (6.77)	34.48 (6.51) ^f^	37.54 (6.66)	37.81 (6.90)	20–35%
Saturated fat (g)	27.27 (10.31)	27.13 (10.91)	25.66 (9.93)	24.06 (9.54) ^f^	28.54 (9.77)	28.93 (10.12)	NA ^c^
Dietary cholesterol (g)	284.55 (92.58)	283.26 (98.51)	269.13 (90.20)	254.96 (86.40) ^f^	295.86 (90.94)	299.30 (93.71)	NA ^d^
**Male (*n* = 100)**							
**Dietary Intake Variables**	**No/Low Attendees (*n* = 33)**		**High Attendees (*n* = 19)**		**Never Enrolled (*n* = 48)**		**Recommended Intakes ^a^**
	**S1**	**S3**	**S1**	**S3**	**S1**	**S3**	
Fruit/vegetable score	9.64 (7.52)	10.18 (7.24)	10.21 (6.52)	8.90 (4.74)	10.65 (5.51)	12.19 (6.93)	NA
Fruit/vegetable/bean score	13.52 (10.36)	15.18 (10.05)	14.53 (8.15)	13.37 (6.73)	15.27 (7.54)	17.46 (10.02)	NA
Meat/snack score	24.76 (13.64)	24.61 (9.91)	22.79 (12.56)	20.79 (11.33)	24.40 (8.81)	25.60 (9.52)	NA
**Fruit and vegetable intake**							
Fruit and Vegetable servings (per day)	3.34 (2.77)	3.54 (2.68)	3.56 (2.39)	3.06 (1.75)	3.71 (2.04)	4.29 (2.55)	
**Micronutrient intake**							
Vitamin C (mg)	132.68 (68.43)	143.45 (67.26)	137.12 (54.96)	129.36 (44.88)	145.18 (49.85)	159.36 (66.62)	90.0
Magnesium (mg)	379.58 (121.17)	398.04 (120.78)	382.88 (100.26)	369.01 (81.40)	403.38 (88.55)	427.80 (118.70)	420.0
Potassium (mg)	3500.18 (1195.21)	3684.40 (1185.90)	3547.87 (978.58)	3411.32 (795.34)	3729.64 (872.31)	3973.00 (1168.72)	3400.0
Dietary fiber (g)	18.38 (8.13)	19.58 (8.19)	18.36 (6.90)	17.43 (5.61) ^f^	20.05 (5.96)	21.65 (7.99)	38.0
**Fat intake**							
Total fat (g)	92.12 (32.73)	91.76 (23.77)	87.40 (30.14)	82.60 (27.18)	91.25 (21.14)	94.15 (22.85)	NA ^b^
Fat %	34.66 (8.18)	34.56 (5.94)	33.47 (7.54)	32.27 (6.80)	34.44 (5.29)	35.16 (5.71)	20–35%
Saturated fat (g)	31.19 (12.00)	31.05 (8.72)	29.45 (11.05)	27.69 (9.97)	30.87 (7.75)	31.93 (8.38)	NA ^c^
Dietary cholesterol (g)	336.40 (114.44)	335.22 (84.35)	332.43 (97.15)	316.83 (87.26)	332.40 (74.27)	341.83 (82.07)	NA ^d^

note. SD = Standard deviation; S1 = baseline survey; S3 = end of program survey; NA = Not applicable. ^a^ NIH, Nutrient Recommendations: Dietary Reference Intakes (DRI), the adequate intake for ages group 31–50. Available from: https://ods.od.nih.gov/HealthInformation/Dietary_Reference_Intakes.aspx (accessed on 9 November 2021). ^b^ Neither an Estimated Average Requirement (EAR), and thus a Recommended Dietary Allowance (RDA), nor an Adequate Intake (AI) was set for total fat for individuals aged 1 year and older because data were insufficient to determine an intake level at which risk of inadequacy or prevention of chronic disease occurs. ^c^ Neither an EAR (and thus an RDA) nor an AI was set for saturated fatty acids because they are not essential and have no known role in preventing chronic disease. ^d^ Neither an EAR (and thus an RDA) nor an AI was set for cholesterol. However, it is recommended that people maintain their dietary cholesterol intake as low as possible while consuming a diet nutritionally adequate in all required nutrients. Institute of Medicine 2006. Dietary Reference Intakes: The Essential Guide to Nutrient Requirements. Washington, DC: The National Academies Press. https://doi.org/10.17226/11537 (accessed on 9 November 2021). ^e^ the value of no/low attendees is significantly different from high attendees at the same time point at *p* < 0.05. ^f^ the value of high attendees is significantly different from never enrolled students at the same time point at *p* < 0.05.

**Table 3:** Household food insecurity prevalence by program enrollment status and level of attendance based on the survey data, mean (SD), or frequency (%).

Characteristic	Analytic Sample (*n* = 385 ^a^)	Analytic Sample (*n* = 385)			Enrolled into the Program (*n* = 251)		
		Never Enrolled in the Program (*n* = 134)	Enrolled into the Program (*n* = 251)	Comparison Never Enrolled vs. Enrolled ^b^	No/Low Attendees (*n* = 172)	High Attendees (*n* = 79)	Comparison No/Low vs. High Attendees ^b^
**Food insecurity at S1 (baseline)**							
Food secure	150 (39.0%)	60 (44.8%)	90 (35.9%)		63(36.6%)	27 (34.2%)	
Food insecure	235 (61.0%)	74 (55.2%)	161 (64.1%)	z = −1.710	109 (63.4%)	52 (65.8%)	z = −0.376
Low food security	117 (30.4%)	41 (30.6%)	76 (30.3%)	z = −0.065	54 (31.4%)	22 (27.9%)	z = 0.568
Very low food security	118 (30.7%)	33 (24.6%)	85 (33.9%)	z = −1.873	55 (32.0%)	30 (38.0%)	z = −0.933
**Food insecurity at S3 (final time point)**							
Food secure	159 (41.3%)	74 (55.2%)	85 (33.9%)		60 (34.9%)	25 (31.7%)	
Food insecure	226 (58.7%)	60 (44.8%)	166 (66.1%)	z = −4.055 ***	112 (65.1%)	54 (68.4%)	z = −0.503
Low food security	89 (23.2%)	25 (18.7%) ^c^	64 (25.5%)	z = −1.517	43 (25.0%)	21 (26.6%)	z = −0.267
Very low food security	137 (35.6%)	35 (26.1%)	102 (40.6%)	z = −2.834 ***	69 (40.1%)	33 (41.8%)	z = −0.248

^a^ number is less than 1000 due to only 39% of students completed both the S1 and S3 surveys. ^b^ comparison test used was probability tests for categorical variables. *** *p* < 0.001 ^c^ The rate at S3 is significantly different from the rate at S1 at *p* < 0.05.

**Table 4:** Main findings from the focus groups: barriers and facilitators associated with FDP utilization.

Barriers	Facilitators
**1.** **Program barriers—design and organization** “The only thing I can say was when you got to the end where they would weigh the food, that could’ve been a little discouraging. Because if you were picking your own bags, or even the bags they give you, or the meat or maybe the dairy, when you get to the end, they put it on a scale, and if it is this and this certain amount, they will take something out. So that could’ve been something that I really need that you took out and you’re telling me…” “Yeah, I had medical issues. I had a couple surgeries. And I had to get a co-worker of mine to go up there with me. Because the check-in process was really long and I wasn’t supposed to be up just period. So just standing there in line waiting.” “I think they had more options in the beginning. Then as time progressed, the options changed, too.” “I think that’s slow, not—I mean I get it like once you check-in it’s find. But I shouldn’t have to check out I believe. I think that kind of slowed things down a little bit because you’re checking in, you go through the line, you get the end, you got to go through the same process.” “And because it was such slim pickings they were sending you through the line three and four times trying to get you to meet that quota on that weight amount.”	**1.** **Program facilitator—type of food and welcoming environment** “But in most cases, we brought our own bags to carry, a cart, or whatever to carry it, but they also had carts that you could put _____ _____ in and take it to your car, and unload it in your car and bring the cart back. So, that was helpful, too, yeah.” “Really, it was really convenient, because when you would get there, they would give you the option of having a bag that they had already packed with each food group. Or you can go through and get what you wanted off the tables from, you know, each food group.’ “Yeah, because there weren’t too many sweets. Like I didn’t see that as far as giving us cakes. So if you don’t have it, you’re not going to consume it, right? So yeah, I didn’t see cakes and stuff. Unhealthy.” “The selections were, the specials were I think good and excellent because we had a choice of everything on the pyramid, of meat, vegetables, different things.” “They treated us equally, they made us feel like we were welcome there, and that’s the biggest thing of it, when somebody’s doing something like this, you know, you want them to feel, like, accepted.”
**2.** **Personal barriers—schedule and transportation** “I sometimes—most of the time, either I had class, I couldn’t leave class and go. And so, this lady that was picking it up for me and taking it home for me, she couldn’t do it no more, so that really knocked me out of the box, ‘cause she couldn’t do it no more. And when I wasn’t in class, that wasn’t the day they’d get it. The day that they’d bring it over there to the Newton, it was my class, I had class.” “And then I found out that it was over here at Newton. Highland didn’t have it, Pin Oak didn’t have it, Saddle Brook didn’t have it. So I had no other option but—and like I said because I work in the Hacienda it was kind of convenient when I was at work. But coming from home, I’m passing up two other campuses to get there.” “My location for sure. Only because I live in Wilcox and at the time, I didn’t have a car, which is why online classes was really easy for me but that would have been my only obstacle, actually getting to the food bank.” “Well, first of all, I work part time and I go to school part time, so the timing affects me, because I also ride Metro. I’m a seizure patient, so I don’t try to drive. My seizures are basically controlled, but I do have problems with stress, and pressure, and have seizures occasionally, so I ride Metro. So basically, my biggest I guess you can say problem would be timing.” “Transportation, yeah, _____ _____ challenge, ‘cause it’s kind of difficult, by the time you get home, the meat is thawed out and, you know, so you got to cook it ‘cause it’s already thawed out. So now you got to _____ _____ _____ cook it, you can’t put it back in the fridge, and…” “Yeah, they missed it, because I live in Katy; if I didn’t have a car, there’s no bus that comes in, where I live in Kollen, to over here. So, yeah, there’s a lot of people that may have classes here, but they live over here and there’s no public transportation to get you from Point A to Point B. And everybody can’t afford Uber or Lyft or going to pay somebody to take them somewhere, you know? They only have maybe the resources to get them to that specific class at that location at that time, yeah, so.”	**2.** **Program facilitator—Substitute shopper** “I lived so close to campus, and I also had, like, housemates that, you know, one of my housemates was willing to go and stand in and pick up for me.” “And it was me and my daughter and my daughter would go for me because during that time I had surgery. Yeah, and I wasn’t supposed to be out.” “And so if you weren’t able to go, you could send someone to go for you, and I think it was usually two different locations” “Usually the challenges that I had was regarding time and I would just send my son in my place to go and get it. So when they were having it either I was at work or I was in class, so I would just let him go. If I didn’t have him, then it probably would have been more of a challenge, but he was able to compensate for where I couldn’t go.” “And also one thing other, they had a part of it if you weren’t able to make it you could send someone. So that was really good”
**3.** **Program barrier—Program abuse by other attendees** “Because people were being greedy, getting too much. Then that became a problem.” “I can’t really say. No, it was a good variety of everything that time that I went. At the beginning, I was kind of in the middle of the line, so I noticed that a lot of people were trying to—I think the limit was 50 pounds or something, so a lot of people were just trying to get meat and that was not leaving a good selection for the people that were behind them if you only grabbed meat and nothing else.” “It came at a time where I really needed it and I don’t have anything negative to say about the program. It’s just some of the people, I think, were discourteous and they went in with an attitude like, “Hey, I’m going in with me and my daughter. I’m going to bring my daughter with me and me and my daughter’s going to get this.” And I’ve seen some people get eight, nine, ten packs of meat, and I was, wait. This is crazy.” “People on your back, people reaching over your shoulder, or you’re trying to get your products, and they see something they want, so they just go around you and grab it. That was my only kickback about it, and I’m the type of person, I’m going to let you go ahead and get what you want.”	

## Supplementary Materials

The following are available online at www.mdpi.com/article/10.3390/ijerph182212106/s1, Supplementary Figure S1: Consort diagram of cohort 1, Supplementary Figure S2: Consort diagram of cohort 2, Supplementary File S1: Dietary intake calculations, Supplementary Table S1: Demographic characteristics of the students in two FDP cohorts (*n* = 2000) and bivariate comparison between cohort 1 (*n* = 1000) and cohort 2 (*n* = 1000). Given as mean (SD) or frequency (%).
