# Supplementary material for: Feasibility of Delivering an on-Campus Food Distribution Program in a Community College Setting: A Mixed Methods Sequential Explanatory Investigation
Source: Int J Environ Res Public Health. 2021 Nov 18;18(22):12106. doi: 10.3390/ijerph182212106 (PMC8619067; doi:10.3390/ijerph182212106)
Supplement: Supplementary file 1 [file ijerph-18-12106-s001.zip › ijerph-1425058-supplementary.pdf]

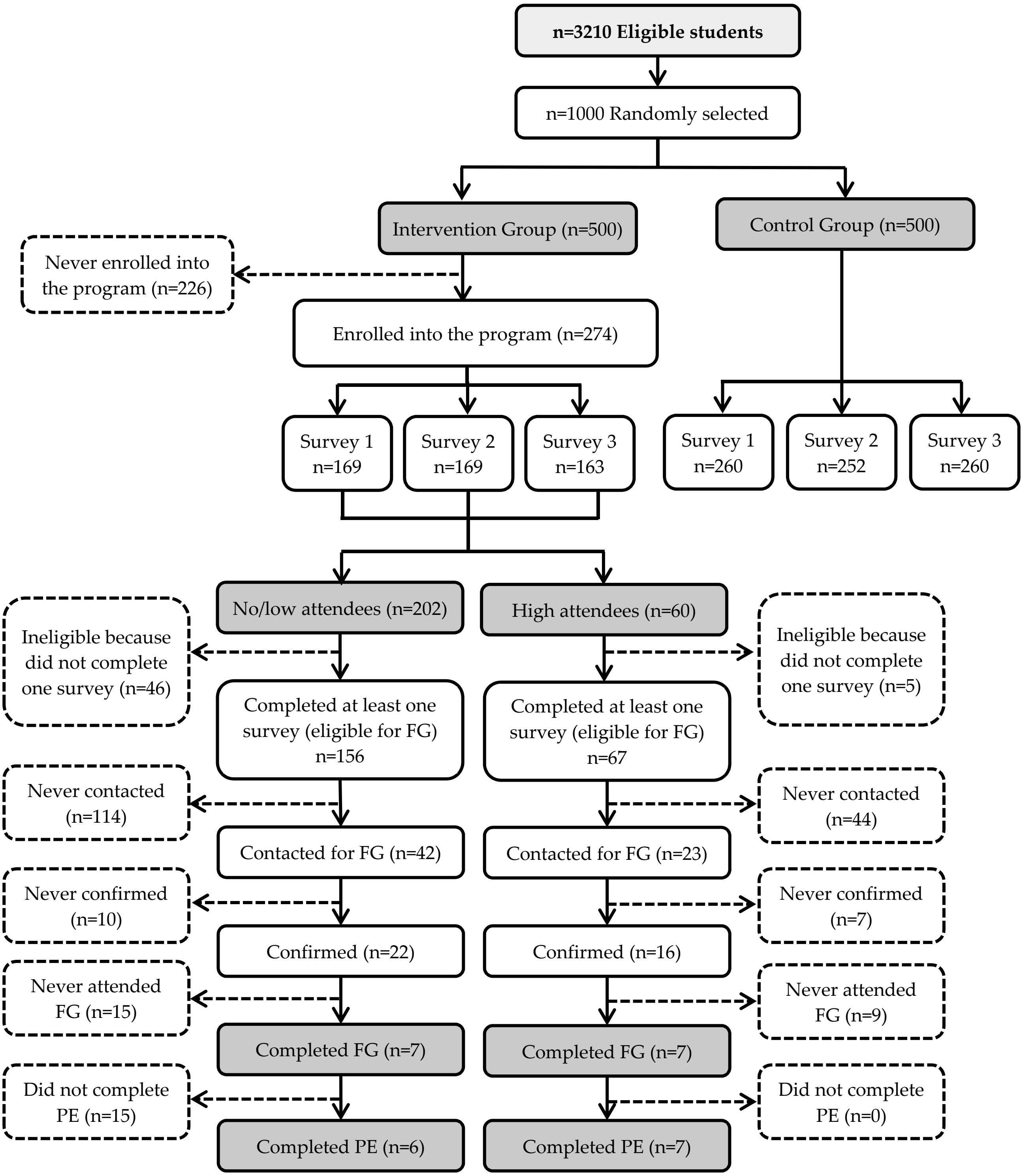

Supplementary Figure S1. Consort diagram of cohort 1
No/low attendees= attended 0-2 food distributions; High attendees= attended ≥3 food distributions; FG= Focus group; PE=Photo elicitation
Note. Those who did not complete at least one survey were ineligible for focus groups
---- Excluded from the sample.

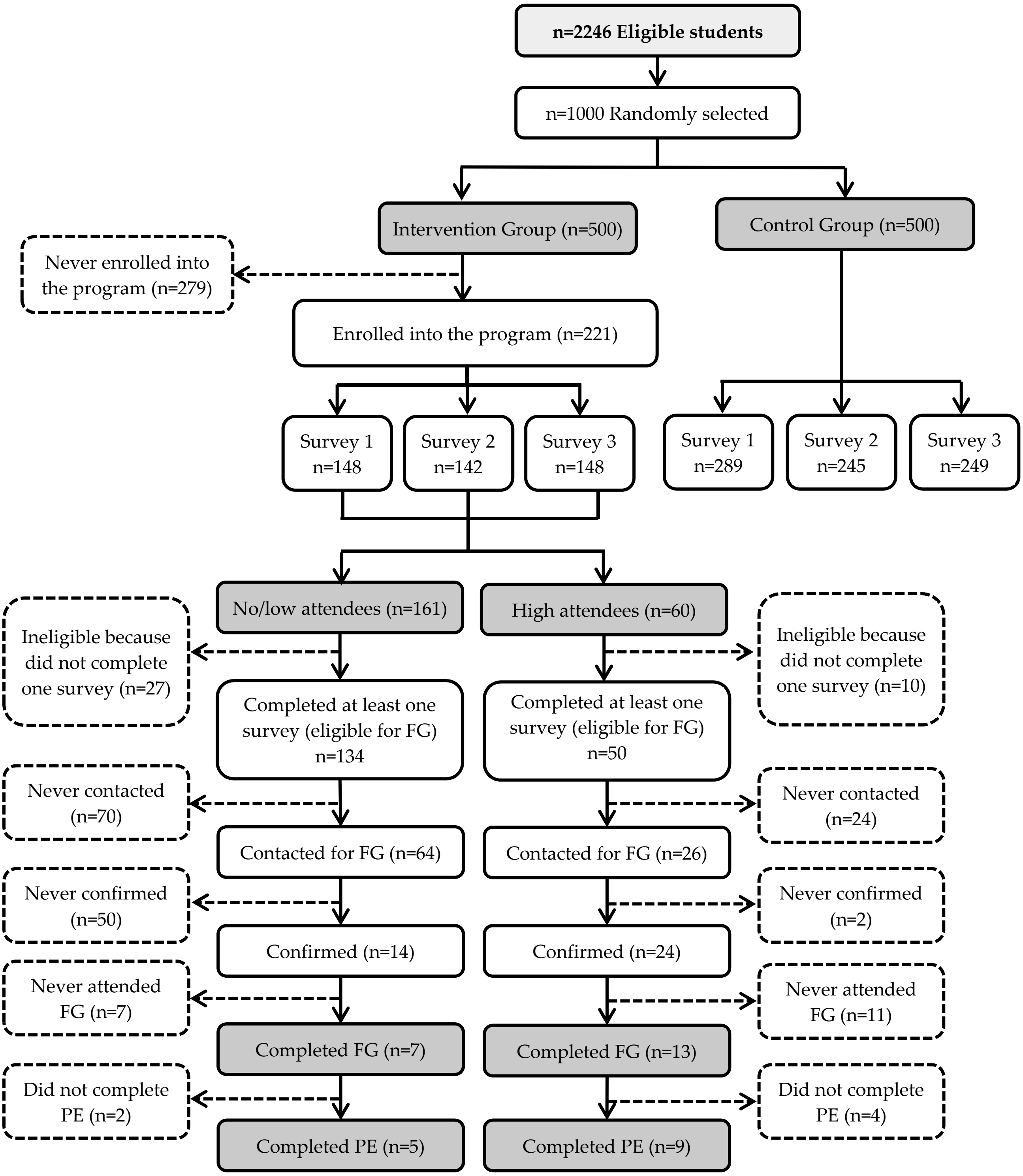

Supplementary Figure S2. Consort diagram of cohort 2
No/low attendees= attended 0-2 food distributions; High attendees= attended ≥3 food distributions; FG= Focus group; PE=Photo elicitation
Note. Those who did not complete at least one survey were ineligible for focus groups
---- Excluded from the sample.

Supplementary File S1. Dietary intake calculations

Block Fruit-Vegetable-Fiber Screener. The Fruit-Vegetable-Fiber screener includes 10-items to capture fruit and vegetable intake, fiber, and micronutrients found in fruits and vegetables. Respondents are asked to consider their eating habits over the past year regarding: fruit juice, fresh/canned fruit, vegetable juice, green salad, potatoes, vegetable soup/stew, other vegetables (e.g., broccoli, string beans), fiber cereals, beans (baked beans, lentils), and dark bread (e.g., whole wheat or rye) based on a 0=*Less than once a week*; to 5= *2 or more times a day scale*. The responses were summed to create a fruit/vegetable score (7 items) and a fruit/vegetable/beans score (10 items). The following equations were used to calculate the fruit/vegetable servings and the estimates of Vitamin C, magnesium, potassium, and dietary fiber [26].

Fruit/vegetable servings per day = - 0.23 + [0.37 * (Fruit/Vegetable score)] - [0.55 * Sex]	**[26]**
*Vitamin C (mg) = 56.5 + [6.6 * (Fruit/Veg/Beans score)] - [26.7 * Sex] - [0.45 * Age]*	[26]
*Magnesium (mg) = 272 + [11.6 * (Fruit/Veg/Beans score)] - [92.3 * Sex] - [1.7 *Age]*	[26]
*Potassium (mg) = 2348 + [114.8 * (Fruit/Veg/Beans score)] - [759 * Sex] - [13.8 * Age]*	[26]
*Dietary Fiber (g) = 12.6 + [0.77 * (Fruit/Veg/Beans score)] - [5.12 * Sex] - [0.16 * Age]*	[26]

Note: Negative values that were derived from the calculations above were recoded to zero.

Block Meat/Snack Screener. The Meat/Snacks screener includes 17-items to capture dietary fats. Respondents were asked to consider eating habits in the past year regarding: hamburgers/tacos, beef/pork/ribs, fried chicken, hot dogs/sausage, cold cults/ham, bacon/breakfast sausage, salad dressing, margarine/butter, eggs, pizza, cheese/cheese spread, whole milk, French fries, potatoes chips/popcorn/crackers, doughnuts/cake, cookies, and ice cream. Response options are on a 1 = *Once a month or less* to 5 = *5 or more times a week* scale. The meat/snack score was the sum of responses. The following equations were used to calculate the estimate of total fat, saturated fat and dietary cholesterol [26].

Total fat (g) = 32.7 + [2.4 * (Meat/Snack score)] + [11.2 * Sex]	**[26]**
*Saturated fat (g) = 9.4 + [0.88 * (Meat/Snack score)] - [3.5 * Sex]*	[26]
*Percent fat = 19.8 + [0.6 * (Meat/Snack score)] + [2.3 * Sex]*	[26]
*Dietary cholesterol (g) = 120 + [7.8 * (Meat/Snack score)] - [54.65 * Sex] + [36.6 * Race]*	[26]

Note: Negative values that were derived from the calculations above were recoded to zero.

Supplementary Table S1. Demographic characteristics of the students in two FDP cohorts (n=2000) and bivariate comparison between cohort 1 (n=1000) and cohort 2 (n=1000). Given as mean (SD) or frequency (%).

Characteristic	Total sample (n=2000)	Cohort 1 (n=1000)	Cohort 2 (n=1000)	Comparison test statistics	
				t value/ Chi^2 value	P value
**Age**	29.54 (10.23)	29.40 (9.85)	29.68 (10.61)	t= -0.612	0.541
**Gender**					
Male	661 (33.1%)	335 (33.5%)	326 (32.6%)	Chi^2=0.183	0.669
Female	1,339 (67.0%)	665 (66.5%)	674 (67.4%)		
**Race/ethnicity**					
Non-Hispanic white	155 (7.8%)	80 (8.0%)	75 (7.5%)	Chi^2=0.175	0.676
Non-Hispanic black	1,135 (56.8%)	581 (58.1%)	554 (55.4%)	Chi^2=1.485	0.223
Hispanic	559 (28.0%)	263 (26.3%)	296 (29.6%)	Chi^2=2.704	0.100
Other	155 (7.6%)	76 (7.6%)	75 (7.5%)	Chi^2=0.007	0.933
**Marital status**					
Married	142 (7.1%)	77 (7.7%)	65 (6.5%)	Chi^2=1.092	0.296
Divorced/separated	152 (7.6%)	66 (6.6%)	86 (8.6%)	Chi^2=2.870	0.090
Single	1,705 (85.3%)	857 (85.7%)	848 (84.8%)	Chi^2=0.265	0.607
**Academic level**					
Freshman	1,449 (72.5%)	722 (72.2%)	727 (72.7%)	Chi^2=0.063	0.802
Sophomore	117 (5.9%)	63 (6.3%)	54 (5.4%)	Chi^2=0.735	0.391
Associate degree	152 (7.6%)	69 (6.9%)	83 (8.3%)	Chi^2=1.396	0.237

Bachelor's degree	50 (2.5%)	26 (2.6%)	24 (2.4%)	Chi^2=0.082	0.775
Master's degree	10 (0.5%)	5 (0.5%)	5 (0.5%)	Chi^2=0.000	1.000
Unclassified/not available	222 (11.1%)	115 (11.5%)	107 (10.7%)	Chi^2=0.324	0.569
**Employment status**					
Full-time employee	224 (11.2%)	112 (11.2%)	112 (11.2%)	Chi^2=0.000	1.000
Part-time employee	277 (13.9%)	135 (13.5%)	142 (14.2%)	Chi^2=0.205	0.650
Not employed	1,499 (75.0%)	753 (75.3%)	746 (74.6%)	Chi^2=0.131	0.718
**Cumulative GPA**					
At study enrollment	2.45 (1.02)	2.42 (1.02)	2.48 (1.03)	t = -1.220	0.223

Note. Data is based on the student-level administrative data provided by the community college. Percentages may not total 100 due to rounding. The comparison test reported includes independent sample *t*-test for continuous variable and Chi square tests for categorical variables.
